# Novel biomarkers in acute kidney injury: their role in the diagnosis of kidney dysfunction and etiology definition, their potential as predictive markers of structural renal damage severity

**DOI:** 10.1007/s11739-026-04362-6

**Published:** 2026-05-04

**Authors:** Gaetano Pacinella, Maria Grazia Basso, Giuseppe Miceli, Alessandra Casuccio, Mario Daidone, Stefania Scaglione, Federica Todaro, Chiara Pintus, Giuliana Rizzo, Antonino Tuttolomondo

**Affiliations:** 1 Department of Health Promotion, Mother and Child Care, Internal Medicine and Medical Specialties (ProMISE), Università Degli Studi Di Palermo, Palermo, Italy; 2 Internal Medicine and Stroke Care Ward, G. D’, Alessandro University Hospital, Policlinico Paolo Giaccone, Palermo, Italy

**Keywords:** Acute kidney injury, AKI, Renal disease, Novel biomarkers, Kidney dysfunction, Acute renal disease, KIM-1, PENK, IGFB*P-*7, TIM*P-*2, Nephrocheck, NGAL, LFABP, CYR61

## Abstract

Acute kidney injury (AKI) is a life-threatening condition whose early diagnosis is crucial. The most used method to evaluate renal function is the glomerular filtration rate (eGFR). The detection of new circulating molecules has gained traction for the early identification of kidney damage. In this prospective observational study, 57 patients with acute kidney disease and 23 patients without acute renal damage were consecutively enrolled; urinary concentrations of NGAL, LFABP, CYR61, TIM*P-*2, IGFB*P-*7, and [TIM*P-*2 X IGFB*P-*7], and serum concentrations of PENK and KIM-1 were obtained in all patients. The primary endpoint was to assess the role of serum and urinary markers in distinguishing prerenal from renal pathogenesis of AKI. The secondary endpoint was to evaluate the possible association between urinary and serum concentrations of these markers and the severity of acute kidney injury. Urinary TIM*P-*2, NGAL, and IGFB*P-*7 concentrations were higher in patients with AKI compared with the control group, with statistical significance. Among patients with AKI, we found higher concentrations of LFABP, Cyr61, TIM*P-*2, NGAL, IGFB*P-*7, and [TIM*P-*2]x[IGFB*P-*7] according to AKI aetiology, with statistical significance maintained in multivariable logistic regression for IGFB*P-*7. The ROC curve confirmed that IGFB*P-*7 has a predictive role in the aetiological diagnosis of AKI. A significant association between urinary LFABP and TIM*P-*2 and serum KIM-1 concentrations (*p* = 0.0001) and the variation in creatinine values from baseline to enrollment was found. Furthermore, we found a statistically significant correlation between KIM-1 and the variation in creatinine levels from admission to discharge. This study highlights an association between the concentrations of the novel biomarkers and the aetiology of AKI, with a possible role for these molecules in stratifying patients with acute renal disease.

## Background

Acute kidney injury (AKI) is defined as an abrupt and often reversible nephron dysfunction that impairs the elimination of metabolic products and the maintenance of acid–base and electrolyte balance. Diagnostic criteria include an increase in serum creatinine of 0.3 mg/dL within 48 h, a ≥ 50% increase within seven days, or urine output < 500 mL for more than six hours [1].

According to the underlying aetiology, AKI can be classified as prerenal, intrinsic, or postrenal. Prerenal causes are related to reduced effective circulating volume or renal hypoperfusion; intrinsic AKI results from structural damage to glomeruli or tubules; and postrenal AKI is due to urinary tract obstruction, leading to increased hydrostatic pressure and impaired filtration.

Several classification systems, including the RIFLE and AKIN criteria, have been developed to stratify disease severity, mortality risk, and therapeutic needs [2, 3]. AKI is highly prevalent, accounting for 8–16% of hospitalisations [4], with incidence ranging from 2–5% in general wards to up to 67% in intensive care units [5]. Early diagnosis is essential to prevent progression to chronic kidney disease and reduce mortality.

Renal function is commonly assessed by estimating glomerular filtration rate using serum creatinine–based equations such as Cockcroft–Gault, CKD-EPI eGFRcr, and CKD-EPI eGFRcys. However, serum creatinine does not allow reliable differentiation between prerenal and intrinsic causes of AKI, potentially delaying diagnosis and treatment. Although surrogate markers such as cystatin C are available, no biomarkers are routinely used in clinical practice to stratify AKI aetiology and guide personalised management.

In recent decades, several circulating molecules have emerged as promising tools for early AKI detection, including fatty acid binding proteins (FABPs) [10–12],  CYR61, TIM*P-*2, IGFB*P-*7, NGAL, PENK, and KIM-1. Urinary LFABP has shown earlier increases than serum creatinine and diagnostic performance comparable to NGAL, with AUC values generally between 0.70 and 0.85 [13]. CYR61 has been investigated in kidney disease, although its diagnostic value in AKI remains less well defined than that of established biomarkers [16].

The combined biomarker [TIM*P-*2] × [IGFB*P-*7], commercially available as NephroCheck, has demonstrated predictive ability for moderate-to-severe AKI, with an AUC of approximately 0.80 in the multicentre Sapphire study [19]. NGAL is one of the most extensively studied AKI biomarkers, with meta-analyses reporting AUC values typically ranging from 0.70 to 0.90 across clinical settings [21]. Plasma PENK has been shown to correlate closely with measured glomerular filtration rate and may provide earlier functional assessment than creatinine, with performance comparable to cystatin C [23]. Circulating KIM-1 has also been investigated as a marker of structural kidney damage and adverse renal outcomes [26].

Although the literature has attributed heterogeneous roles to these biomarkers—including early diagnostic indicators of AKI, mediators of acute tubular injury, and predictors of renal outcomes—our study specifically aimed to evaluate their performance in early etiological discrimination of AKI. In particular, we investigated whether these circulating and urinary molecules could help distinguish between prerenal and intrinsic renal injury at an early stage of kidney dysfunction, a distinction that remains challenging using conventional markers such as serum creatinine. Within this framework, we also explored the potential prognostic role of KIM-1 by assessing its association with recovery from acute kidney injury. This dual approach allowed us to examine not only the diagnostic contribution of these biomarkers in the etiological classification of AKI but also the possible prognostic value of KIM-1 in predicting renal recovery.

### AIMS of the study

Our study aimed to identify an association between newly identified circulating markers of renal damage and the pathogenic mechanisms of acute kidney injury. The primary endpoint was to assess whether serum and urinary markers can distinguish prerenal from renal pathogenesis of AKI.

The secondary endpoint was to evaluate whether urinary concentrations of NGAL, LFABP, CYR61, TIMP2, IGFBP7, and [TIM*P-*2 X IGFB*P-*7], as well as serum concentrations of PENK and KIM-1, were associated with the severity of acute kidney injury. Severity was estimated by delta creatinine, defined as the difference between admission and baseline or discharge serum creatinine values.

## Materials and methods

In this prospective observational study, we consecutively enrolled 57 patients with AKI and 23 patients without AKI, all admitted for diseases other than acute kidney injury (including syncope, ischemic stroke, and exacerbated COPD), whose comorbidity profiles were comparable to the AKI group. Inclusion criteria for both groups required age- and sex-matching, normal serum creatinine and blood urea levels (serum creatinine: 0.6–1.3 mg/dL; blood urea: 15–40 mg/dL), and absence of neoplastic, rheumatological, or chronic inflammatory diseases. Exclusion criteria included alcohol abuse, immunomodulatory or immunosuppressive drug use, and steroid therapy. Patients were consecutively admitted to the Internal Medicine and Stroke Care ward at University Hospital "Paolo Giaccone" of Palermo from October 2020 to October 2023.

Following patient enrollment, all participants were informed in detail about the aims, procedures, potential risks, and expected benefits of the study. Written informed consent was obtained from each participant prior to enrollment, in accordance with institutional policies and the ethical principles outlined in the 2001 Declaration of Helsinki. The study protocol was approved by the Ethics Committee of the University Hospital “Policlinico Paolo Giaccone”.

The sample size estimation was based on the expected difference in renal injury biomarker levels between groups, while individual biomarkers were analysed as exploratory outcomes. For sample size calculation, G*Power software (version 3.1.9.7; Heinrich-Heine-Universität Düsseldorf, Germany) was used to perform an a priori power analysis for comparing two independent groups on continuous outcomes (test family: t tests; statistical test: difference between two independent means). Assuming a two-sided α level of 0.05, a statistical power of 80%, and an expected 40% difference in biomarker levels between groups based on previously reported variability in renal injury biomarkers (coefficient of variation approximately 0.32), the required sample size was estimated as 18 patients per group. To account for a potential 5% dropout rate, the total planned sample size was set at 36 patients. Because multiple biomarkers were evaluated, analyses of individual markers were considered exploratory, and results were adjusted for multiple comparisons using the Benjamini–Hochberg false discovery rate procedure.

Although the calculated minimum sample size was 36 patients, all eligible patients admitted during the predefined study period were consecutively enrolled, thereby increasing the robustness and precision of the statistical analyses. As a result, the final sample represents the total number of patients who met the inclusion criteria during the study period.

To accurately identify AKI among participants, we applied the KDIGO (Kidney Disease: Improving Global Outcomes) classification, defining AKI as the presence of one of the three following criteria: an increase in serum creatinine of more than 0.3 mg/dL within 48 h or more than 50% within one week or contraction of the urine volume < of 0.5 mL/Kg/h for at least six hours.

Patients underwent a medical history assessment for comorbidities and risk factors (Table 1), physical examination, and routine blood tests after 8 h of fasting. Previous basal and discharge serum creatinine values were collected. Urinary and serum biomarker concentrations were measured. Blood samples, collected after fasting, were centrifuged and stored at − 80 °C. Biomarkers were quantified by commercial ELISA kits. Biochemical analysis was conducted by the laboratory at University Hospital "Paolo Giaccone" in Palermo.

**Table 1:** Comparison of the urinary and serum biomarker levels between the study population and healthy controls

Marker	Patients with AKI (*n* = 57)	Healthy controls (*n* = 23)	*p*
LFABP (median, IQR) (pg/mL)	3.7 (2.3–16)	4.0 (1.9–16.9)	0.962
CYR61 (median, IQR) (pg/mL)	107 (51.6–303.1)	96 (57–217)	0.510
TIM*P-*2 (median, IQR) (pg/mL)	1.4 (0.7–4.8)	1.3 (0.6–2.3)	0.172
NGAL (median, IQR) (pg/mL)	13.3 (5.2–20.7)	6.4 (3.2–10.6)	**0.009**
IGFB*P-*7 (median, IQR) (ng/mL)	453 (210–2108)	221 (103–337)	**0.011**
TIM*P-*2 X IGFB*P-*7 (median, IQR) (ng/mL)	812 (284–3288)	306 (73–1058)	**0.044**
PENK (median, IQR) (pmol/L)	1866 (1518–2507)	2950 (2207–4435)	** < 0.0005**
KIM-1 (median, IQR) (pg/mL)	0.22 (0.04–0.7)	0.23 (0.1–1.1)	0.573

*FABP1* fatty acid binding protein 1, *CYR61* urinary cysteine-rich protein 61,*NGAL* Neutrophil gelatinase associated lipocalin,*IGFBP-7* Insulin-like growth factor-binding protein 7, *TIMP-2* tissue inhibitor of metalloproteinase-2,*PENK* proenkephalin-A,*KIM-1* Kidney injury molecule-1

*P* = raw *p-*value

Statistically significant values (with *p* 0.05) are reported in bold so that readers can identify them more easily among all the data

In line with standard diagnostic criteria, we further classified enrolled patients with AKI based on aetiology: pre-renal AKI was defined as increased serum azotaemia, absence of abnormalities in the urine sample, and FeNa (Fractional Excretion of Sodium) less than 1% [27, 28]; conversely, diagnosis of intrinsic AKI was made in the presence of FeNa > 1%, normal azotaemia, and medical history suggestive of glomerular or tubular disease, vasculitis, or drug-induced damage [29].

However, FeNa is known to have important limitations, particularly in patients receiving diuretic therapy, as diuretics can increase urinary sodium excretion, thereby reducing the diagnostic reliability of this index. Given this challenge, it is notable that in this study, fractional excretion of urea (FeUrea), which is considered more reliable in patients treated with diuretics, was not routinely measured. Consequently, the classification of AKI aetiology may be subject to potential misclassification bias in patients exposed to diuretics.

Building upon this etiological classification, we have investigated differences in biomarker concentrations in serum and urinary samples from recruited patients with prerenal or intrinsic kidney injury to assess the possible role of these markers in the etiological diagnosis of AKI

**Figure:**
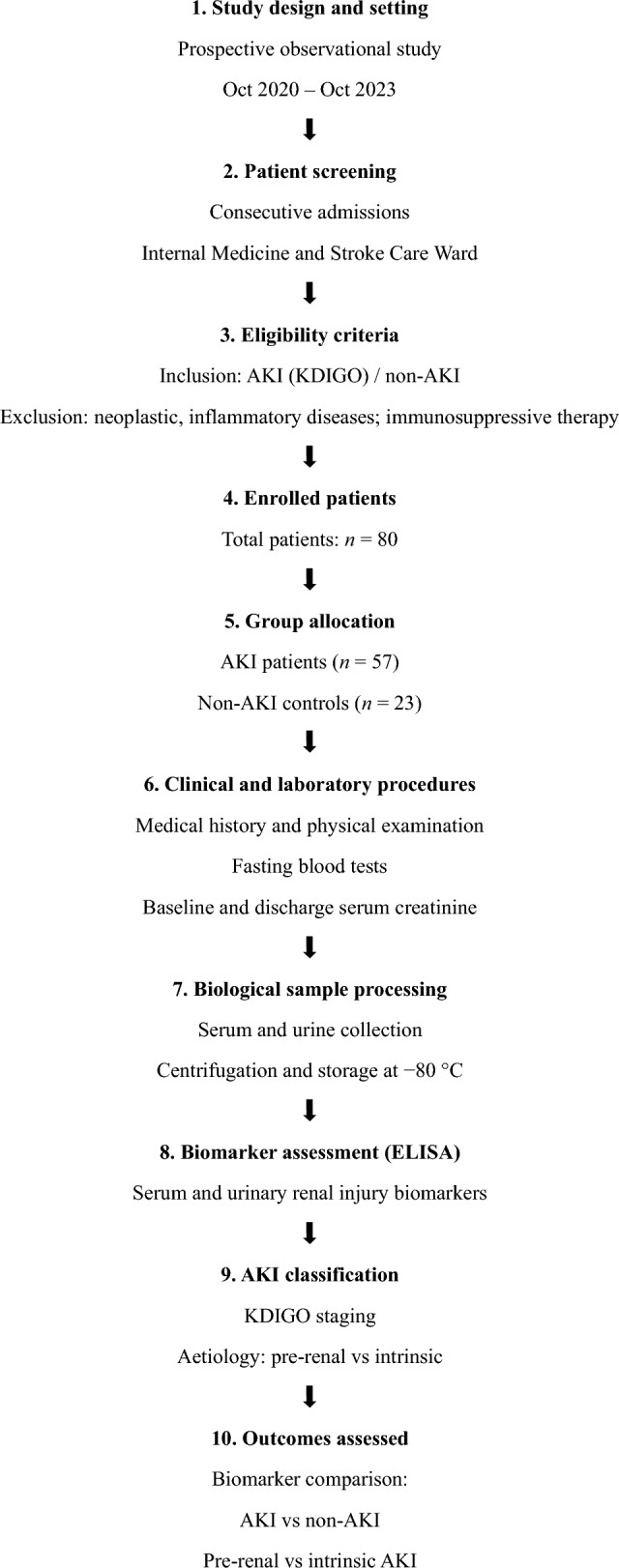

### Data analysis

Quantitative and qualitative data were analysed using descriptive statistics. Continuous variables are presented as mean ± standard deviation (SD) for normally distributed data and as median with interquartile range (IQR) for non-normally distributed variables. Normality of continuous variables was assessed using the Shapiro–Wilk test. Comparisons between groups were performed using one-way analysis of variance (ANOVA) for normally distributed variables and the Mann–Whitney U test for non-normally distributed variables. Categorical variables were analysed using Pearson’s chi-square test or Fisher’s exact test, as appropriate.

To account for multiple testing across the analysed biomarkers, the false discovery rate (FDR) was controlled using the Benjamini–Hochberg procedure with a threshold of *q* = 0.05.

We used a multivariable logistic regression model to examine associations between serum renal injury biomarkers (independent variables) and patient groups (dependent variable). Results are reported as odds ratios (OR) with 95% confidence intervals (CI). We selected candidate variables based on biological relevance and prior links to acute kidney injury. We assessed model fit with the Hosmer–Lemeshow goodness-of-fit test.

We used Spearman’s correlation coefficient and a two-sided t-test to evaluate the correlation between markers’ serum concentrations and changes in creatinine values from baseline to enrollment.

To assess the predictive performance of different cut-offs of IGFB*P-*7 serum levels across patient groups, we performed receiver operating characteristic (ROC) analysis, calculating the area under the curve (AUC) and 95% CI, and reporting sensitivity and specificity.

The *p-*values reported in the text and tables correspond to raw (unadjusted) values obtained from the statistical tests. In the multivariable logistic regression analysis, *p-*values correspond to the raw *p-*values of the model coefficients. To account for multiple testing across the analysed biomarkers, the false discovery rate (FDR) was controlled using the Benjamini–Hochberg procedure, and the corresponding adjusted q-values are reported where appropriate. Data were analysed using IBM SPSS Statistics 24 (IBM Corp., Armonk, NY, USA). All *p-*values were two-sided, and p ≤ 0.05 was considered statistically significant.

The STRING database was used to analyse protein–protein associations by identifying potential interaction partners and visualising interaction networks.

## Results

We enrolled 57 patients diagnosed with AKI and 23 patients without AKI.

The AKI patient group consisted of 20 females and 37 males, with a mean age of 75 years (74.2 ± 7.9). They were divided into two subgroups based on the aetiology of kidney damage: 33 patients with prerenal AKI and 24 with renal AKI. We reported gender and age data for each subgroup (prerenal and renal AKI) in the AKI group.

Patients in the control group had a similar gender distribution, with 8 women (35% of the total) and 15 men (65% of the total), with an average age of 73.4 years ± 8.3.

Patients with AKI showed significantly higher median urinary levels of NGAL [13.3 (5.2–10.7) vs 6.4 (3.2- 10.6); *p* = 0.009], IGFB*P-*7 [453 (210–2108) vs 221 (103–337); *p* = 0.011], and [TIM*P-*2 X IGFB*P-*7] [812 (284-

3288) vs 306 (73–1058); *p* = 0.044], but they had lower serum levels of PENK compared to the control group, with statistical significance (Table 1). The average length of hospital stay was 7 days for cases and 6 days for controls; the two subgroups did not differ in clinical variables, except for a higher incidence of nephrotic syndrome (*p* = 0.0001) in the renal subgroup and a higher prevalence of diuretics (*p* = 0.028) in the prerenal subgroup. Patients with prerenal AKI were older (*p* = 0.001). At enrollment, patients with renal AKI showed higher creatinine values [3.4 (1.9–5.8) vs 1.9 (0.2–2.9); *p* = 0.015] and more accentuated differences with the baseline values, compared with the prerenal AKI patients [2.2 (1.3–4.1) vs 1.07 (0.7–2.2); *p* = 0.002] (Table 2).

**Table 2:** Descriptive analysis of the population: laboratory variables in patients with prerenal and intrinsic AKI and types of treatment administered during hospitalization for prerenal and intrinsic AKI

Variables	Prerenal AKI *n* (%) = 33 (58%)	Intrinsic AKI *n* (%) = 24 (42%)	*p*
Gender (female/male, *n* (%)	12 (36%)/21 (64%)	8 (33%)/16 (67%)	0.78
Age (years)	74.3 ± 9.8	76.1 ± 10.2	0.42
Red blood cells (mean ± SD) (× 10^6/µL)	3.61 ± 0.76	3.22 ± 0.63	**0.042**
Hematocrit (mean ± SD) (%)	29.52 ± 6.2	27.60 ± 5.9	0.244
Hemoglobin (mean ± SD) (g/dL)	10.96 ± 5.0	9.29 ± 1.91	0.131
Creatinine (median, IQR) (mg/dL)	1.90 (0.2–2.9)	3.4 (1.9–5.8)	**0.015**
Glomerular flow rate (mean ± SD) (ml/min)	22.90 ± 12.2	15.29 ± 11.3	**0.021**
Delta creatinine 1* (median, IQR) (mg/dL)	1.07 (0.7–2.2)	2.2 (1.3–4.1)	**0.002**
Delta creatinine 2° (median, IQR) (mg/dL)	0.99 (0.7–1.5)	1.36 (0.2–2.6)	0.539
Calcium (mean ± SD) (mmol/L)	7.24 ± 3.3	7.48 ± 2.2	0.760
Phosphoremia (mean ± SD) (mmol/L)	3.50 ± 2.5	3.68 ± 1.8	0.767
Sodium (mean ± SD) (mmol/L)	139.51 ± 10.8	138.37 ± 4.9	0.634
Potassium (mean ± SD) (mmol/L)	4.36 ± 0.91	4.46 ± 0.9	0.672
Magnesium (mean ± SD) mmol/L	1.78 ± 0.8	1.74 ± 0.5	0.841
NT-proBNP (median, IQR) (pg/mL)	6090.0 (2683–9363)	5620.5 (3327–9681)	0.877
Ferritin (mean ± SD) (ng/mL)	528.45 ± 74.4	493.333 ± 59.3	0.849
CRP (median, IQR) (mg/L)	66.0 (7.3–147.5)	55.4 (21.8–101.8)	0.663
Lactates (mean ± SD) (mmol/L)	1.71 ± 0.6	1.67 ± 0.9	0.872
Treatment	Prerenal AKI *n* (%) = 33 (58%)	Intrinsic AKI *n* (%) = 24 (42%)	*p*
Loop diuretics, *N*° (%) Yes No	24 (73%) 9 (27%)	20 (83%) 4 (17%)	0.524
Anti-hypertensive drugs, *N*° (%) Yes No	16 (48%) 17 (52%)	15 (62%) 9 (38%)	0.420
Dialysis, *N*° (%) Yes No	1 (3%) 32 (97%)	8 (33%) 16 (67%)	**0.003**
Hydration, N° (%) Yes No	29 (88%) 4 (12%)	22 (92%) 2 (8%)	0.696

*NT-proBNP* N-terminal prohormone of brain natriuretic peptide*,CRP* C-reactive protein

***P* = **raw *p-*value

^*^Delta creatinine 1: difference between the creatinine value at admission and the basal value

°Delta creatinine 2: difference between the creatinine value at discharge and the value at admission

Statistically significant values (with *p* 0.05) are reported in bold so that readers can identify them more easily among all the data

Therapeutic strategies (Table 2) did not differ between subgroups, except for a higher use of dialytic treatment in renal AKI, which was performed in 33% of this subgroup (*p* = 0.003). At the same time, no differences were found in the use of diuretics (*p* = 0.524), hydration (*p* = 0.696), and anti-hypertensive treatment (*p* = 0.420).

Patients with a renal AKI (24 patients) in comparison with ones with a prerenal etiology (33 patients) showed significantly higher serum levels of LFABP [8.0(2.9–59) vs 3.0 (2.1–6.4); *p* = 0.047)], Cyr61 [203 (59–493) vs 94 (44–200); *p* = 0.030], NGAL [18 (12–22) vs 9.8 (4–16); *p* = 0.011], IGFB*P-*7 [1714 (350–2367) vs 234 (128–626); *p* = 0.001)], and [TIM*P-*2xIGFB*P-*7] [2684 (784–9288) vs 360 (177–1261); *p* = 0.002)] (Table 3).

**Table 3:** Comparison between the urinary and serum biomarker levels in patients with prerenal and intrinsic AKI

Marker	Prerenal AKI (*n* = 33)	Intrinsic AKI (*n* = 24)	*p*
LFABP (median, IQR) (pg/mL)	3.0 (2.1–6.4)	8.0 (2.9–59)	**0.047**
CYR61 (median, IQR) (pg/mL)	94 (44–200)	203 (59–493)	**0.030**
TIM*P-*2 (median, IQR) (pg/mL)	1.3 (0.7–3.7)	3.3 (0.7–9.9)	0.110
NGAL (median, IQR) (pg/mL)	9.8 (4–16)	18 (12–22)	**0.011**
IGFB*P-*7 (median, IQR) (ng/mL)	234 (128–626)	1714 (350–2367)	**0.001**
TIM*P-*2 X IGFB*P-*7 (median, IQR) (ng/mL)	360 (177–1261)	2684 (784–9288)	**0.002**
PENK (median, IQR) (pmol/L)	1833 (1510–2325)	2226 (1511–2727)	0.383
KIM-1 (median, IQR) (pg/mL)	0.25 (0.04–0.71)	0.21 (0.03–0.85)	0.948

*FABP1* fatty acid binding protein 1, *CYR61* urinary cysteine-rich protein 61,*NGAL* neutrophil gelatinase associated lipocalin,*IGFBP-7* Insulin-like growth factor-binding protein 7,*TIMP-2* tissue inhibitor of metalloproteinase-2,*PENK* proenkephalin-A, *KIM-1* Kidney injury molecule-1

*P* = raw *p-*value

Statistically significant values (with *p* 0.05) are reported in bold so that readers can identify them more easily among all the data

After controlling for multiple testing with the Benjamini–Hochberg procedure, only IGFB*P-*7 remained statistically significant (*q* = 0.008). Associations for LFABP and TIM*P-*2, which were nominally significant in unadjusted analyses, did not retain significance after FDR correction. (Table 4).

**Table 4:** Multivariable logistic regression analysis for the correlation among the markers of AKI and the etiology of acute renal damage

Biomarkers	Odds Ratios (OR)	95% confidence interval for OR		*P*	q_BH
		Lower limit	Upper limit		
LFABP	**1.386**	**1.043**	**1.841**	**0.025**	0.1
CYR61	1.005	0.998	1.012	0.177	0.241
TIM*P-*2	0.146	0.020	1.073	0.059	0.118
NGAL	1.080	0.957	1.218	0.211	0.241
IGFB*P-*7	**1.002**	**1.001**	**1.004**	**0.001**	**0.008**
TIM*P-*2 X IGFB*P-*7	1.0	1.0	0.99	0.290	0.29
PENK	1.00	1.0	1.01	0.056	0.118
KIM-1	1.595	0.805	3.163	0.181	0.241

*q_BH p-*value adjusted for multiple comparisons using the Benjamini–Hochberg false discovery rate procedure,*FABP1* fatty acid binding protein 1, *CYR61* urinary cysteine-rich protein 61,*NGAL* neutrophil gelatinase associated lipocalin,*IGFBP-7* Insulin-like growth factor-binding protein 7, *TIMP-2* tissue inhibitor of metalloproteinase-2,*PENK* proenkephalin-A, *KIM-1* kidney injury molecule-1

*P*** = **raw *p-*value from the multivariable logistic regression model

Statistically significant values (with *p* 0.05) are reported in bold so that readers can identify them more easily among all the data

Furthermore, we have tried to associate the values of these markers with an improving or worsening as changes of creatinine serum values to establish the severity of kidney damage, defining two variables: delta creatinine 1, which refers to the entity of the renal damage through the evaluation of the difference between the value of the creatinine at admission and the basal value previous the kidney injury, and the delta creatinine 2, defined by the difference between the creatinine value at the admission and the value at the discharge.

We have found a significant correlation using Spearman’s test between the urinary values of LFABP (*p* = 0.006), TIM*P-*2 (*p* = 0.040), and the serum concentration of KIM-1 (*p* = 0.048), with a higher value of the delta creatinine 1, underlining a greater worsening of the creatinine at admission compared with the basal values, and so a more severe harm to renal function. Furthermore, a statistically significant correlation was found between KIM-1 and the positive value of delta creatinine 2, indicating the degree of improvement in renal function, defined as the extent of the reduction in creatinine values at discharge compared with admission (Table 5).

**Table 5:** Spearman’s correlation analysis of the mean values of the biomarkers with the delta creatinine in AKI patients (*N*° = 57)

**Biomarkers**	Delta creatinine 1* (*N* = 57)	**Delta creatinine 2° (*N* = 57)**
LFABP		
*r*	**0.359**	0.133
*p*	**0.006**	0.325
CYR61		
*r*	0.243	0.137
*p*	0.068	0.310
TIM*P-*2		
r	**0.273**	0.091
*p*	**0.040**	0.500
NGAL		
*r*	**0.407**	0.067
*p*	**0.002**	0.618
IGFB*P-*7		
*r*	**0.421**	**0.284**
*p*	**0.001**	**0.132**
TIM*P-*2 X IGFB*P-*7		
*r*	**0.431**	0.225
*p*	**0.001**	0.093
PENK		
*r*	-0.055	-0.173
*p*	0.687	0.198
KIM-1		
*r*	**0.260**	**0.370**
*p*	**0.048**	**0.044**

*FABP1* fatty acid binding protein 1,*CYR61* urinary cysteine-rich protein 61,*NGAL* neutrophil gelatinase associated lipocalin,*IGFBP-7* insulin-like growth factor-binding protein 7, *TIMP-2* tissue inhibitor of metalloproteinase-2,*PENK* proenkephalin-A, *KIM-1* Kidney injury molecule-1

^*^Delta creatinine 1: difference between the creatinine value at admission and the basal value

°Delta creatinine 2: difference between the creatinine value at discharge and the value at admission

Statistically significant values (with *p* 0.05) are reported in bold so that readers can identify them more easily among all the data

ROC curve analysis showed that IGFB*P-*7 had the highest discriminative ability for distinguishing intrinsic from prerenal AKI (AUC = 0.756; 95% CI 0.63–0.87; *p* = 0.001). The optimal cut-off value was approximately 750, with a sensitivity of about 74% and a specificity of about 78%. TIM*P-*2 × IGFBP7, CYR61 and TIM*P-*2 also showed moderate discriminative performance, whereas the other biomarkers did not significantly discriminate between groups: the analysis showed an AUC of 0.740 (95% CI 0.62–0.86; *p* = 0.003) for TIM*P-*2 × IGFB*P-*7, with an optimal cut-off of 1200 (ng/mL)^2^/1000 (sensitivity 0.71, specificity 0.76); CYR61 yielded an AUC of 0.730 (95% CI 0.61–0.85; *p* = 0.004) with a cut-off of 250 ng/mL (sensitivity 0.70, specificity 0.75), while TIM*P-*2 alone showed an AUC of 0.720 (95% CI 0.60–0.84; *p* = 0.006) at a threshold of 3.5 ng/mL (sensitivity 0.69, specificity 0.73); NGAL showed an AUC of 0.620 (95% CI 0.48–0.76; *p* = 0.14) with a cut-off of 15 ng/mL (sensitivity 0.60, specificity 0.63); FABP1 had an AUC of 0.600 (95% CI 0.46–0.74; *p* = 0.20) with a cut-off of 10 ng/mL (sensitivity 0.58, specificity 0.61), and KIM-1 showed an AUC of 0.570 (95% CI 0.43–0.71; *p* = 0.35) at a threshold of 0.30 ng/mL (sensitivity 0.55, specificity 0.60). PENK showed an AUC of 0.420 (95% CI 0.28–0.56; *p* = 0.22).

A combined model including IGFB*P-*7 and TIMP2 × IGFBP7 showed improved discriminative ability (AUC 0.820) compared with IGFB*P-*7 alone (Fig. 2).

## Discussion

This study is focused on the association between urinary markers and the aetiology of kidney damage; we demonstrated a statistically significant association between LFABP, CYR61, NGAL, IGFB*P-*7, and [TIM*P-*2 × IGFB*P-*7] concentrations and the intrinsic aetiology of AKI. Understanding the pathways involving these molecules is essential for interpreting the biological significance of their association with AKI.

From a mechanistic perspective, these biomarkers reflect different pathophysiological pathways involved in AKI. FABPs participate in intracellular lipid trafficking and metabolic regulation [6–9], whereas CYR61 is involved in extracellular matrix signalling processes, including angiogenesis, apoptosis, and fibrosis [14, 15]. TIM*P-*2 and IGFB*P-*7 act as mediators of cell-cycle arrest during early cellular stress, potentially limiting the proliferation of injured tubular cells [17, 18]. NGAL is associated with iron transport and tubular epithelial stress responses [20], while PENK, a freely filtered low-molecular-weight peptide, has been linked to real-time changes in glomerular filtration [22]. KIM-1 contributes to tubular repair mechanisms and phagocytic clearance of cellular debris following injury [24, 25]. These heterogeneous biological functions suggest that combining functional and structural biomarkers may improve AKI phenotyping and risk stratification.

Several studies have shown a strong diagnostic role for LFABP in chronic kidney disease and diabetic nephropathy [28, 29], as well as in acute renal damage, as demonstrated in studies of patients who underwent cardiac surgery and developed ischemia/reperfusion renal injury [30, 31]. In the kidney, LFABP is expressed in the proximal tubule and secreted into the tubular lumen along with other metabolites [32]. LFABP expression is induced by hypoxia through the expression of HIF-1alpha and PPAR-α (peroxisome proliferator-activated receptor-α) [33, 34], so we can assume that its urinary levels should be increased in case of prerenal AKI. However, several studies have demonstrated that the increased concentration of LFABP in the urine derives from the failure of resorption by mechanisms of megaline-dependent endocytosis from the renal cells [35], while less importance is given to the high serum levels [36–38], so the tubular damage promotes the high elimination of this protein in the urine. This could explain the higher concentrations of these markers in the case of intrinsic renal damage. CYR61 is expressed in the Henle loop, in the distal tubule and the collector duct. Moreover, antibodies against CYR61 reduce levels of proinflammatory cytokines [39–41], suggesting a potential role for this molecule in proinflammatory pathways. The involvement of CYR61 in mechanisms of cellular death suggests that this protein could be a marker of structural injury, with urinary concentrations peaking at 6–9 h and normalising within 24 h of the injury [42, 43].

The Nephrocheck’s diagnostic and prognostic value in AKI is supported by large clinical studies [44–47].

The early detection of acute kidney injury is crucial for prompt treatment. After ischemic injury, the tubular epithelium releases NGAL into the urine, earning it the title of the "troponin" of the kidney [48]. Circulating macrophages are then recruited and accumulate in the renal interstitium [49], further perpetuating the damage process. In our study, we confirm the diagnostic role of some of these molecules: we found that NGAL, IGFB*P-*7, and [TIM*P-*2 X IGFB*P-*7] concentrations were statistically higher in patients with AKI than in controls, underscoring the potential use of these markers for detecting renal injury, as demonstrated in previous studies [22]. Notably, these molecules offer earlier diagnostic potential than eGFR; the early increase in acute kidney injury is key to recognising and treating it promptly.

Moreover, evaluating these diagnostic biomarkers could identify the underlying pathophysiologic mechanism of renal injury. Only IGFB*P-*7 remained statistically significant (*q* = 0.008 associated with the AKI setiology); associations for LFABP and TIM*P-*2, which were nominally significant in unadjusted analyses, did not retain significance after FDR correction.

**Fig. 1:**
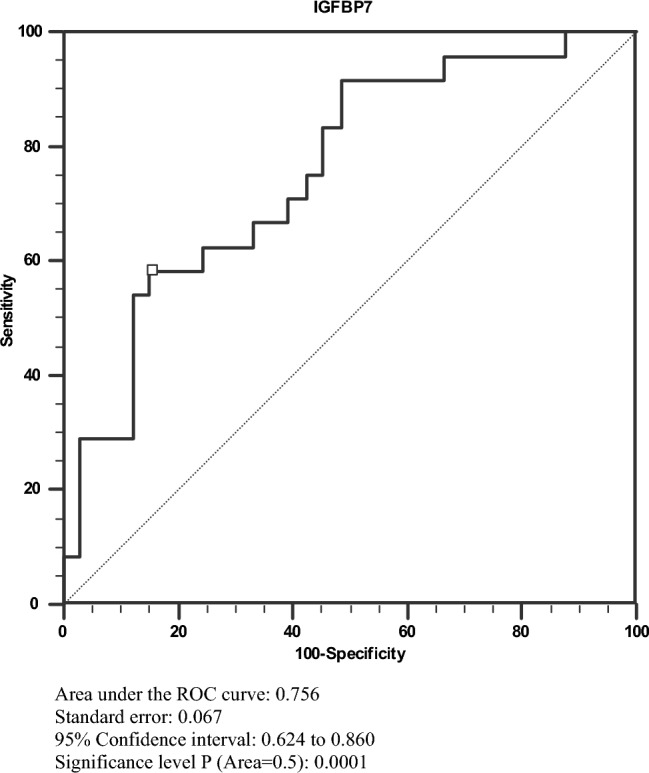
ROC (Receiver Operating Characteristic curve) curve and AUC (Area Under the Curve) for IGFBP7 in the differential diagnosis between prerenal and renal aetiology. Area under the ROC curve: 0.756. Standard error: 0.067. 95% Confidence interval: 0.624–0.860. Significance level *P* (Area = 0.5): 0.0001

The use of the ROC curve allowed us to graphically report that IGFB*P-*7 has a role in the etiologic diagnosis of AKI, since, with an AUC of 0.756 and p < 0.001, it takes on the task of directing the diagnostic pathway of patients who present to the physician’s attention with acute kidney damage that has not yet been well identified, and consequently directing therapy in relation to the type of kidney injury (Fig. 1).

**Fig. 2:**
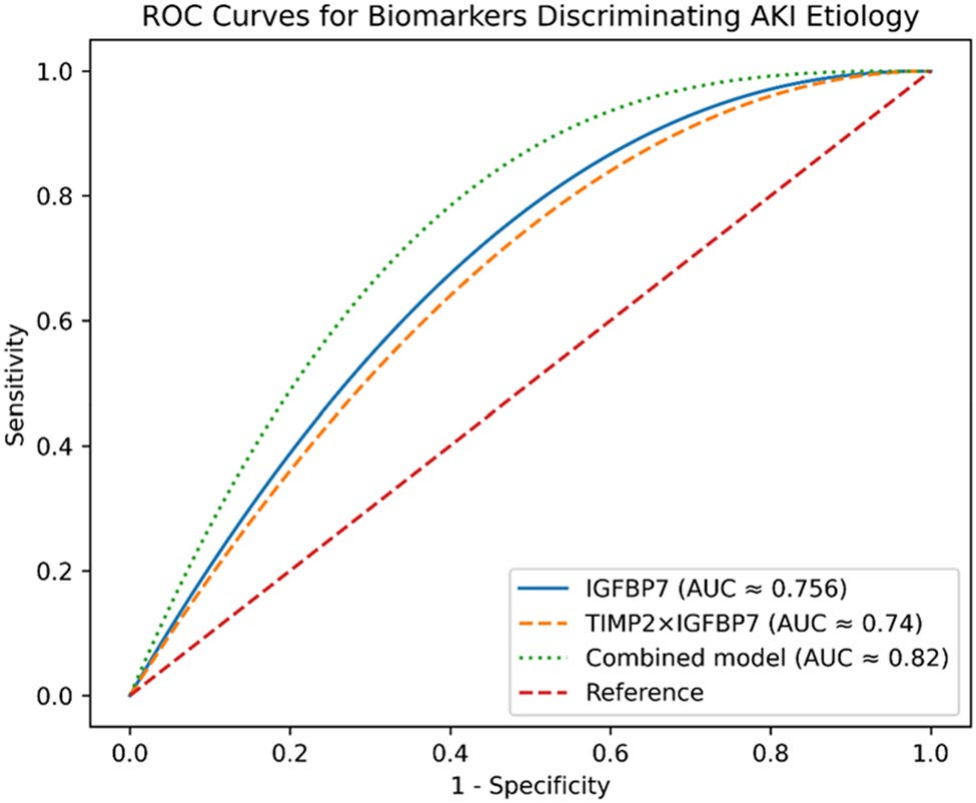
Receiver operating characteristic (ROC) curves showing the discriminative performance of IGFB*P-*7, TIMP2 × IGFBP7 and their combined model for distinguishing intrinsic from prerenal acute kidney injury

TIM*P-*2 × IGFBP7, CYR61, and TIM*P-*2 also demonstrated moderate discriminative performance, whereas the other evaluated biomarkers did not significantly differentiate between groups. Notably, the combined model including IGFB*P-*7 and TIM*P-*2 × IGFBP7 further improved overall accuracy, indicating that a multimarker approach may enhance risk stratification compared with the use of a single biomarker (Fig. 2). However, these findings must be interpreted with caution, as they derive from a relatively small sample size and therefore should be considered purely exploratory. Consequently, the observed discriminative performances and the apparent benefit of the combined model require confirmation in larger, adequately powered cohorts before any firm clinical implications can be drawn.

This premise, if confirmed by subsequent studies, gains relevance in clinical practice: patients with kidney damage should not all be considered the same at the time of first contact with the physician, and therapy for them should not always be the same; early detection of etiology means early action on the cause by counteracting the pathophysiological mechanism of the renal impairment, and rapid patient-tailored therapy toward increasingly personalized precision medicine.

Furthermore, our study has found an association between some of these biomarkers and creatinine variations, defined as delta creatinine 1 and delta creatinine 2. In particular, urinary LFABP, TIM*P-*2, and serum KIM-1 levels were associated with a greater worsening of the renal function (Table 5). In fact, patients with AKI who had higher serum creatinine values at admission, which differed more from basal values, had higher concentrations of LFABP and TIM*P-*2 in the urine and KIM-1 in the serum, supporting a possible role of these proteins in defining the severity of renal injury.

As is well known, these proteins have been recognised as playing a key role in identifying the population at risk of developing AKI. In fact, LFABP has an excellent predictive role in identifying the population at risk of developing acute renal injury since its concentrations increase in urine three days before the rise in creatinine values [50], with a sensitivity of 94,2% and a specificity of 87% of cut-off values of 12.5 μg/g [51] and the PRESERVE trial showed that plasmatic levels of KIM-1 had a good predictive role in selecting the population at risk of AKI after the administration of contrast agent, in which higher levels were detected before the angiographic study, suggesting the possible use of this protein in selecting population with higher risk of developing kidney damage [52]. Tubular renal cells express KIM-1 after ischemic/reperfusion injury, toxic or inflammatory injury, with increased urinary levels of KIM-1 after injury. KIM-1 has been proposed as a diagnostic biomarker of AKI, with sensitivities and specificities of 86 and 74%, respectively [27].

However, none of these molecules has previously been attributed a role in stratifying the population by the severity of the damage, as we propose. Moreover, as found in previous studies, we confirm the protective role of KIM-1 in preventing the worsening of renal dysfunction. In support of this hypothesis, we have found higher concentrations of KIM-1 in the serum of patients who had a greater reduction of the creatinine values compared with the values at the time of the acute damage, and, so, it may be related to defining the severity of the renal injury, but also a higher probability of recovery from renal dysfunction.

We could attribute this association to KIM-1’s role in phagocytosis and to reduced recruitment of proinflammatory cells into the tubule after injury, thereby preventing progression toward chronic renal dysfunction: compared to the markers we have discussed so far, we can therefore consider attributing a prognostic role to KIM-1, as it would allow us to estimate the extent of renal damage recovery and thus stratify patients not only on the basis of acute damage but also on the possibility of regaining and restoring renal function.

KIM-1 expression promotes the apoptotic bodies and reduces the recruitment of proinflammatory cells. Although KIM-1 plays a protective role after acute injury, its constitutive expression in the tubular epithelium leads to gradual fibrosis and progression towards chronic nephropathy [53, 54]. Even if it has been demonstrated that macrophages secreting NGAL play a protective role in the progression of renal disease, reducing inflammation and fibrosis and promoting tubular cell proliferation, we have not found an association between NGAL levels and recovery from AKI.

Our finding of higher PENK levels in controls than in patients with AKI should be interpreted with caution. PENK reflects early changes in glomerular filtration, and its concentration may vary with the temporal dynamics of kidney injury, rising shortly after an acute decline in renal function and potentially decreasing during the recovery phase. Therefore, differences in sampling timing may partly explain the observed pattern, as some AKI patients may have been enrolled during a phase of functional recovery. However, this interpretation remains speculative. In addition, the relatively small sample size of the present study (57 AKI patients and 23 controls) may have increased inter-individual variability and contributed to the unexpected distribution of PENK values between groups.

### Interpretation of molecular interactions through the string network

**Fig. 3:**
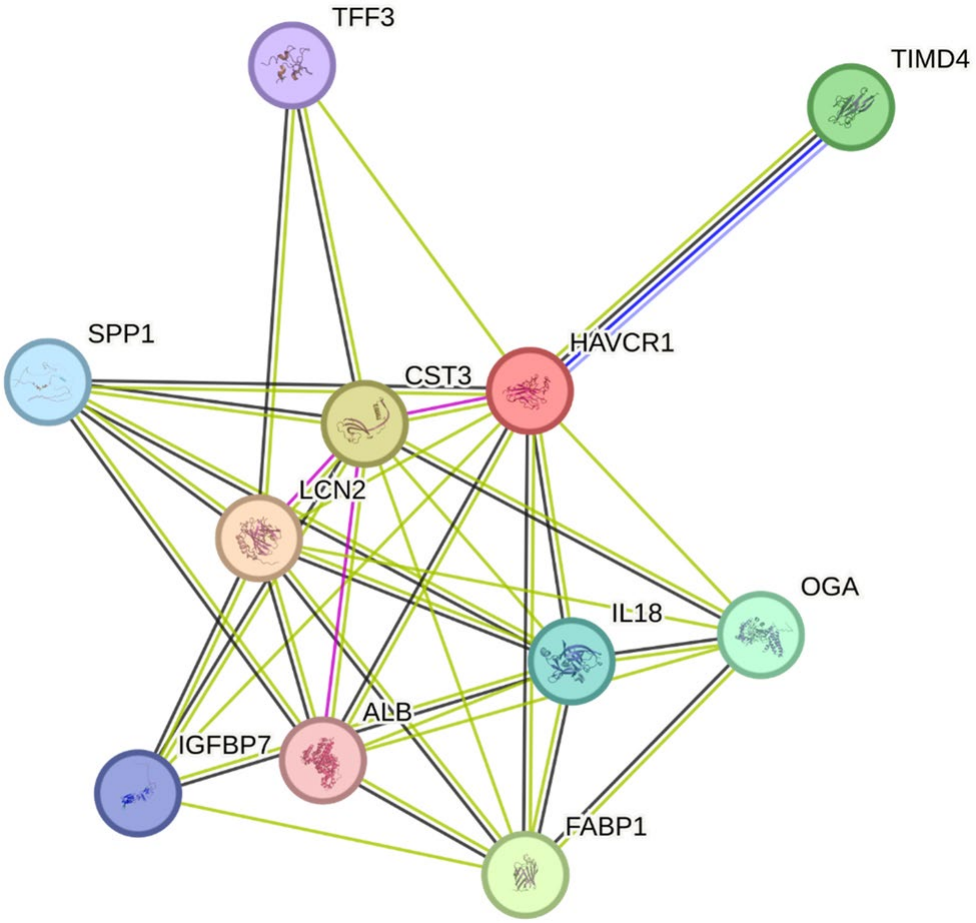
Protein–protein interaction network of the analyzed AKI biomarkers generated using STRING database. *IGFBP7* insulin-like growth factor binding protein 7, *LCN2 (NGAL)* lipocalin-2 (neutrophil gelatinase-associated lipocalin), *HAVCR1 (KIM-1)* hepatitis A virus cellular receptor 1 (kidney injury molecule-1), *FABP1* fatty acid binding protein 1,IL18 interleukin-18, *SPP1* secreted phosphoprotein 1 (osteopontin), *CST3* cystatin C, *ALB* albumin, *TFF3* trefoil factor 3, *TIMD4* T-cell immunoglobulin and mucin domain containing 4, *OGA* O-GlcNAcase

To explore the biological relevance of the results, we used the STRING database to analyse interactions among the molecules investigated. STRING integrates known and predicted protein–protein associations derived from experimental data, computational predictions, and curated databases, providing a comprehensive view of functional relationships within molecular networks. By mapping the selected molecules in the STRING network, we sought to assess whether the observed patterns were supported by known or predicted biological interactions. This approach allowed us to contextualise our results within established molecular pathways and evaluate their potential biological plausibility. The integration of serum and urinary biomarkers with protein–protein interaction analysis confirms that renal AKI is characterised by a distinct molecular signature compared with the prerenal state (Fig. 3).

First, among all biomarkers analysed, IGFB*P-*7 emerged as the most robust indicator of renal parenchymal damage. It was significantly increased in AKI patients compared with controls, further elevated in renal AKI compared with prerenal AKI, and remained the only marker to retain statistical significance after correction for multiple testing. Furthermore, its ROC curve demonstrated fair discriminatory power for etiological classification. From a biological perspective, the interaction network showed that IGFB*P-*7 is centrally located, with multiple connections to key proteins involved in tubular stress and impairment, including LCN2 (NGAL), HAVCR1 (KIM-1), LFABP, and albumin. This centrality supports the concept that IGFB*P-*7 reflects cell cycle arrest and early tubular stress, integrating signals from distinct damage pathways and accounting for its superior statistical performance.

Secondly, LFABP and other biomarkers, such as TIM*P-*2 and CYR61, were significantly higher in renal AKI in unadjusted analyses, and LFABP was also associated with the initial increase in creatinine, suggesting a relationship with the severity of tubular damage. However, these associations did not persist after correction for multiple testing. Network analysis provides a biological explanation for this finding: LFABP is embedded in a larger group of metabolic and inflammatory interactions, suggesting that it reflects general tubular stress rather than a specific aetiological mechanism. Therefore, although LFABP may serve as a marker of damage severity, it appears less specific for distinguishing prerenal from intrinsic renal damage.

Thirdly, KIM-1 showed a dual association with worsening and improving renal function, as evidenced by its correlation with both creatinine delta parameters. This observation is consistent with its biological role. In the interaction network, HAVCR1 (KIM-1) is a highly connected node linking inflammatory mediators, such as IL-18, and markers of structural injury, such as NGAL (LCN2). This pattern supports the interpretation of KIM-1 not only as a marker of tubular injury, but also as an indicator of the repair and remodelling processes occurring during recovery.

Fourthly, inflammation-related proteins, such as IL-18, SPP1 (secreted phosphoprotein 1, also known as Osteopontin), and CST3 (cystatin C), formed a connected subgroup in the network, reflecting shared pathways of inflammation and structural damage. Although these markers contributed to the overall biological context of AKI, they did not retain independent statistical significance after correction for multiple testing, suggesting that they represent common downstream responses rather than aetiology-specific discriminators.

Overall, the integration of clinical data with protein interaction analysis indicates that renal AKI is characterised by a coordinated molecular response centred on cell cycle arrest, tubular stress, and inflammatory signalling. Within this network, IGFB*P-*7 appears to act as a central integrative biomarker, explaining its robustness after statistical correction and its ability to discriminate between prerenal and renal AKI. These findings support the potential clinical utility of IGFB*P-*7 for early aetiological stratification of AKI in emergency settings.

It is important to emphasise that the network-based approach adopted in this analysis was primarily intended to generate biologically plausible hypotheses rather than to establish causal relationships among the investigated molecules. The STRING database integrates heterogeneous sources of evidence, including predicted and indirect associations, which reflect functional connectivity within molecular systems but do not demonstrate direct mechanistic links. Therefore, the observed patterns of interaction should be interpreted as supportive of potential shared pathways and coordinated responses in AKI, providing a framework for future mechanistic and prospective studies aimed at formally testing causal relationships.

### Limitations

Our study has several limitations. Correlation does not mean causation, so we can only assume a pathophysiological link between inflammatory molecules and the cause of renal dysfunction. Limited funds prevented us from enrolling more patients or confirming these results in larger groups. We plan to continue this research, recognising that the small study size may affect the results. This was a single-centre observational study with consecutive patient enrollment. We used statistical methods (e.g., multivariable logistic regression), but a larger sample would yield more robust results; therefore, generalisability must be interpreted with caution. Given the small sample size and the number of biomarkers in our analysis, model overfitting may be a concern. Therefore, interpret the regression model as exploratory and hypothesis-generating, not as a definitive predictive model.

A key limitation of this study lies in the definition of AKI aetiology. The classification of prerenal and intrinsic AKI relied mainly on clinical evaluation and FeNa values. However, FeNa may be unreliable in patients on diuretics, as these drugs increase urinary sodium excretion, possibly leading to misinterpretation of renal sodium handling. FeUrea is considered a more reliable marker in such cases, but was not available in our data. Consequently, the absence of FeUrea measurements may have introduced misclassification bias in AKI categorisation, which should be taken into account when interpreting our results.

## Conclusion

In conclusion, our study offers further suggestions for the possible use of these novel biomarkers for AKI diagnosis in clinical practice. However, the results are from a relatively limited sample and primarily serve as a starting point for considering the data’s implications. These findings will need validation on larger cohorts. Given their diagnostic and predictive roles in acute renal dysfunction, these molecules could help identify populations at risk for AKI and detect renal injury, even before increases in serum creatinine or cystatin C levels, as proposed in earlier studies. Notably, early differential diagnosis of AKI based on aetiology could allow for individualised therapy from the outse.

Moreover, since these molecules are linked to both the severity of renal injury and the likelihood of recovery, data from this study could provide a basis for using these biomarkers to classify AKI patients into risk groups. They could also serve as predictive markers for persistent renal damage rather than recovery. Further studies are needed to support these hypotheses, as this study is exploratory and does not provide conclusive or definitive data.

## Data Availability

All data relevant to the study are included in the article.
